# Anti-DENV-2 Activity of Ethanolic Extracts from *Arachis hypogaea* L.: Peanut Skin as a Relevant Resource of Bioactive Compounds against Dengue Virus

**DOI:** 10.3390/plants13202881

**Published:** 2024-10-15

**Authors:** Florencia Menis Candela, Elio Andrés Soria, Melina Vanesa Moliva, Agostina Suárez Perrone, Elina Beatríz Reinoso, Walter Giordano, María Carola Sabini

**Affiliations:** 1 Departament of Microbiology and Inmunology, Virology Area, Universidad Nacional de Río Cuarto, Ruta 36 Km 601, Río Cuarto CP 5800, Argentina; florenciamcandela@gmail.com (F.M.C.); agostinasuarez13@gmail.com (A.S.P.); 2 Instituto de Investigaciones en Ciencias de la Salud (INICSA), Consejo Nacional de Investigaciones Científicas y Técnicas, Boulevard de la Reforma y Enfermera Gordillo Gómez, Ciudad Universitaria, Córdoba Capital CP 5016, Argentina; easoria@fcm.unc.edu.ar; 3 Instituto de Biología Celular, Facultad de Ciencias Médicas, Universidad Nacional de Córdoba, Boulevard de la Reforma y Enfermera Gordillo Gómez, Ciudad Universitaria, Córdoba Capital CP 5016, Argentina; 4 Departament of Microbiology and Inmunology, Microbial Genetics Area, Universidad Nacional de Río Cuarto, Ruta 36 Km 601, Río Cuarto CP 5800, Argentina; mmoliva@exa.unrc.edu.ar (M.V.M.); ereinoso@exa.unrc.edu.ar (E.B.R.); 5 Instituto de Biotecnología Ambiental y Salud, INBIAS-Consejo Nacional de Investigaciones Científicas y Técnicas, CONICET, Ruta 36 Km 601, Río Cuarto CP 5800, Argentina; giordanow@gmail.com

**Keywords:** *Arachis hypogaea* L., cytotoxicity, antiviral, dengue virus serotype 2, caffeic acid, linoleic acid

## Abstract

Dengue is an emerging disease of high impact on human health. Plants are an important source of new antivirals and *Arachis hypogaea* stands for its biological properties. The aim of this study was to evaluate the cytotoxicity and antiviral activity and elucidate the antiviral mechanism of ethanolic extracts from *A. hypogaea* against dengue virus 2 (DENV-2). The skin or tegument ethanolic extract (TEEs) and seed ethanolic extract (SEEs) were obtained. Cytotoxicity was evaluated by MTT and Neutral Red Uptake (NRU). Antiviral activity was evaluated at different stages of the viral replication cycle by the lysis plaque reduction method. The 50% inhibitory concentration (IC_50_) and selectivity index (SI) were determined. Antiviral activity was further determined by RT-qPCR. The CC_50_ values were 169 (NRU) and 65 (MTT) µg/mL for TEE. In addition, the CC_50_ values were >1400 (NRU) and 636 (MTT) µg/mL for SEE. The TEE demonstrated 99.9 ± 0.1% viral inhibition. The TEE presented an IC_50_ = 3.47 and SI of 48.7 (NRU) and 18.73 (MTT). Its mechanism of antiviral action is broad and it acts in the viral adsorption–penetration stage and inhibits the first steps of infection in the post-penetration stage. It is also capable of acting as virucidal and as prophylactic. Studies of RT-qPCR indicated that the TEE inhibited viral RNA synthesis. These findings suggest that the TEE from *A. hypogaea* could be a promising antiviral candidate for treating DENV-2 infections.

## 1. Introduction

Infection produced by the dengue virus (DENV) represents an emerging disease with a high impact on human health. This flavivirus is a member of the Flaviviridae family. There are four serotypes, DENV-1, DENV-2, DENV-3, and DENV-4, that are transmitted to humans by *Aedes aegypti* and *Aedes albopictus* mosquitoes. A fifth variant (DENV-5) that follows the sylvatic cycle was discovered in October 2013 [1].

DENV is an RNA virus of positive sense, linear, non-segmented, and of approximately 11 kilobases. It encodes a single polypeptide, which is processed in the endoplasmic reticulum by cellular and viral proteases to release structural proteins (C nucleocapsid protein, M membrane-associated protein, and E envelope protein) and non-structural proteins (NS1, NS2A, NS2B, NS3, NS4A, NS4B, and NS5) [2].

The DENV infection in humans ranges from an asymptomatic infection or self-limited febrile illness, called fever dengue, to severe forms, such as dengue hemorrhagic fever, which impairs hemostasis increasing vascular permeability. They also can evolve into dengue shock syndrome in some lethal cases [3]. The rapid expansion of large urban centers, absence of adequate policies for water management, poor housing, viral propagation through human travels, and inefficient vector control programs are among the factors that explain the re-emergence of this disease [4]. DENV-2 has certain disadvantages compared to others, such as its stronger association with severe disease and cross-immunity issues. In addition, its genetic variability complicates vaccine development, treatment options, and epidemiological surveillance [5].

Antiviral drugs are not available for dengue treatment. The only treatment for dengue disease consists of supportive therapies to reduce the consequences of fever, dehydration, hypotension, and bleeding [6]. Therefore, there is an urgent need for effective therapies against DENV. In this regard, plants are important and accessible potential sources of new antiviral drugs [7,8].

An antiviral agent can act by masking the viral proteins E and M necessary for adsorption and entry into the host cell or by blocking intracellular replication [9]. In addition, it can mask viral receptors on the cell surface, due to competitive binding [10]. Other attractive targets are the different cycle steps of viral replication where the agents interfere with non-structural proteins [6]. In recent years, there has been a strong tendency to use plant-based products to treat and prevent health problems [11]. Some plants with antiviral activity against dengue virus have been described [12,13].

*Arachis hypogaea* L., known as the peanut plant, is a legume that belongs to the Fabaceae family. It is native to South America and highly cultivated around the world [14]. Argentina is one of the main producers, which implies an elevated skin (seed coat) discharge as industrial waste that can be recovered [15,16].

This plant stands out for its several scientifically proven biological properties, such as antimicrobial, anti-inflammatory, and antioxidant properties [17,18,19]. In vivo studies indicate the absence of genotoxicity and low toxicity of the peanut skin or tegument (TEE) and seed (SEE) extracts [20]. Chemical analyses reveal potential virucidal molecules, such as proanthocyanidins and phenolic acids, which include caffeic acid in TEE and fatty acids in SEE [20].

Therefore, the current work aimed to evaluate the cytotoxicity and antiviral activity of these extracts against dengue virus serotype 2, which is one of the most aggressive genotypes worldwide. It was based on our hypothesis that peanuts have chemical compounds capable of inhibiting the multiplication of the dengue virus, exerting action in some of the stages of the viral replication cycle.

## 2. Results and Discussion

### 2.1. Cytotoxicity

**Figure 1:**
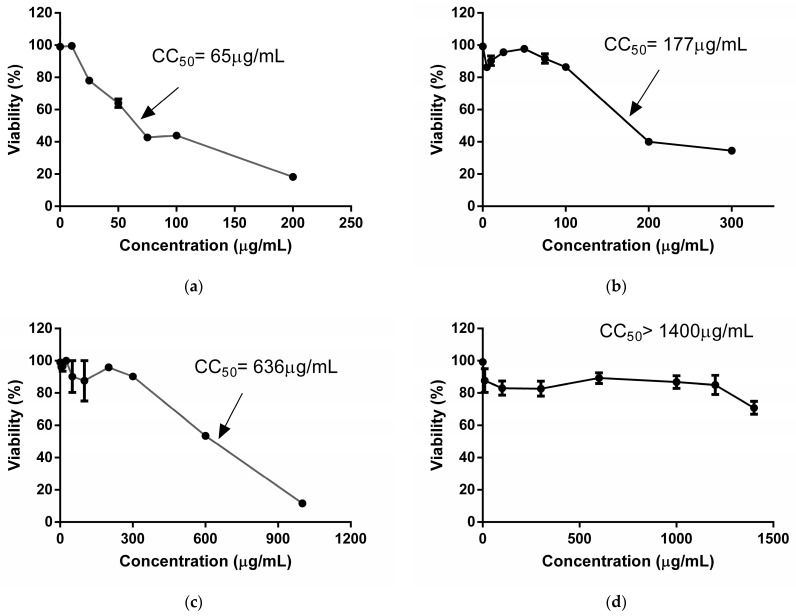
Curves of Vero cell viability treated with the TEE (**a**,**b**) and SEE (**c**,**d**) from *Arachis hypogaea*, assessed by MTT reduction (**a**–**c**) and Neutral Red Uptake (**b**–**d**) after 7 days of treatment.

The results about the cytotoxic activity of extracts are shown in Figure 1. The TEE reduced cellular viability to 50% at lower concentrations than the SEE. Therefore, the TEE demonstrayed greater toxicity than the SEE against Vero cells at 7 days of treatment. With the MTT technique, both extracts showed lower CC_50_ values than those obtained by the NRU technique. This suggested that the TEE and SEE exerted higher damage on mitochondria than lysosomes of these cells. Nonetheless, both extracts are cytotoxic at high concentrations.

Among the scarce studies about the cytotoxicity of *A. hypogaea*, Cossetin et al. (2019) [21] used peanut leaf hydroalcoholic extracts to demonstrate the absence of cytotoxicity in human peripheral blood mononuclear cells. In the same way, Rossi et al. (2020) [16] reported viability percentages of 60% at 500 µg/mL for a peanut skin polyphenolic extract on Vero cells at 24 h of treatment.

Similarly, aqueous extracts of peanut skin, subjected to two digestion steps (P1 and P2), showed low toxicity at 48 h of treatment, with CC_50_ values of 9.4 mg/mL and 15.9 mg/mL in an intestinal cell model (HCT116 cell) [22].

Other authors studied the cytotoxic effect of fractions of an ethanolic extract of peanut skin on melanoma and colorectal cancer cells. These studies indicated toxicity for two fractions when treating the cells with 50 µg/mL for 72 h and determined by MTT [23]. This greater toxicity may be due to the fact that they are fractions of the crude extract.

A previous study of our group revealed that the TEE (300–1600 µg/mL) decreased Vero cellular viability to 60% after 2 days of treatment using the NRU assay. Therefore, the CC_50_ exceeded 1600 μg/mL, whereas the TEE CC_50_ was 600 μg/mL using the MTT assay. SEE CC_50_ values were 1600 µg/mL and >1400 µg/mL after 2 days of treatment using NRU and MTT, respectively [20]. Consequently, prolonged exposure (2 days vs. 7 days) to these extracts increased cytotoxicity, with SEEs being safer.

### 2.2. Antiviral Activity

The viral inhibition percentage for each extract was calculated at different stages to determine if the antiviral activity was due to (i) the induction of an antiviral state in the host cell that prevented subsequent infection (pre-treated cells), (ii) virucidal action by direct contact between extract components and DENV-2 particles that were inactivated and lost their infectivity (pre-treated viruses), (iii) an inhibitory effect at the stage of viral adsorption and penetration or (iv) an inhibitory effect at the stage of viral post-adsorption and penetration. The experimental results are summarized in Table 1, using non-cytotoxic extract concentrations.

The TEE (at 30 µg/mL) displayed antiviral properties in the four stages evaluated. It inhibited DENV-2, with percentages ranging between 84.0 ± 1.4% and 99.9 ± 0.1% (*p* < 0.001). Therefore, the TEE affected the intracellular stages of viral multiplication and promoted an antiviral state in Vero cells protecting them against subsequent infection. Furthermore, it interfered with viral adsorption–penetration and was virucidal. In contrast, the SEE (at 300 µg/mL) did not exert powerful antiviral activity in the stages evaluated since it presented viral inhibition percentages lower than 52%, with the SEE moderately interfering after penetration (intracellular replication or DENV-2 exocytosis).

If two extracts are compared, the TEE has a greater capacity to inhibit the dengue virus, since it can act in all stages tested and block viral production effectively. Both extracts exert the greatest antiviral action in the viral post-adsorption and penetration stage but at different concentrations, i.e., TEE at 30 µg/mL vs. SEE at 300 µg/mL.

### 2.3. Antiviral Activity throughout the Viral Replication Cycle

**Figure 2:**
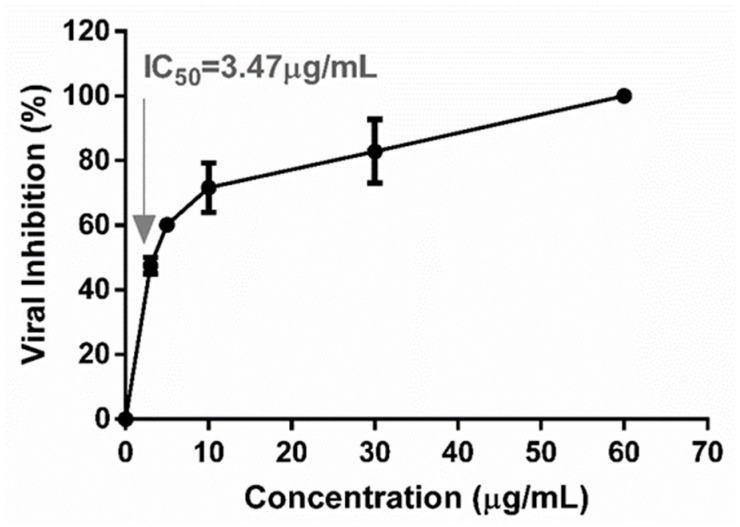
Dose–response curve of DENV-2 inhibition in post-adsorption and penetration by TEE from *Arachis hypogaea* to determine the concentration of extract that inhibits 50% of viral infection (IC_50_). The arrow indicates the value IC_50_.

A dose–response curve was plotted and is shown in Figure 2 with the percentages of viral inhibition obtained from each extract concentration. It can be observed that increasing the TEE concentrations enhanced inhibition. The IC_50_ value of 3.47 μg/mL was obtained by interpolation, with a coefficient of determination greater than 0.90 (R2 = 0.96), which validated the outcome. This IC_50_ was related to the CC_50_ determined by MTT and NRU assays to obtain selectivity indices (SIs) (Table 2).

SI is a measure of the safety margin of an active principle. A greater SI reflects a greater margin (values near to 1 are dangerous). In this study, the SI values were high, which supported TEE use as a selective antiviral drug with a wide margin to be applied on eukaryotic cells.

### 2.4. Quantitative Real-Time PCR Assay (qRT-PCR)

**Figure 3:**
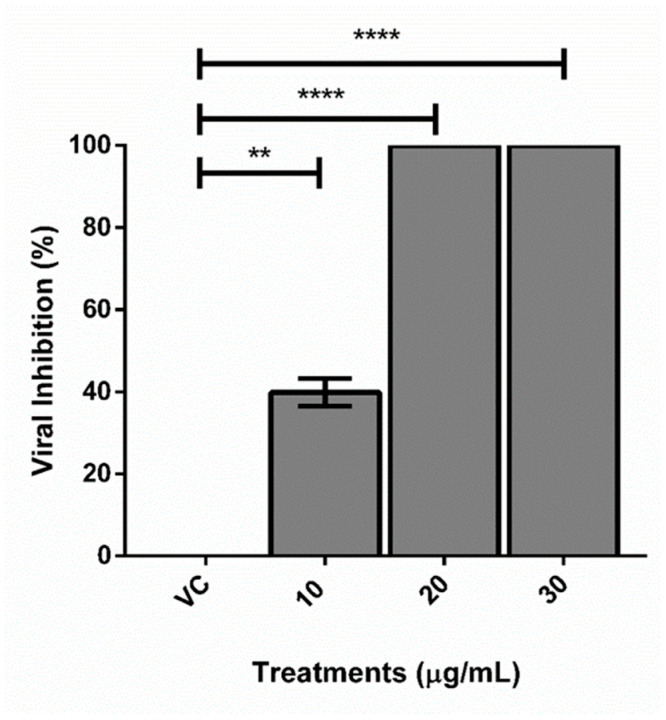
Inhibition of DENV-2 virus (%) treated with TEE from *Arachis hypogaea* at different concentrations (10, 20, and 30 µg/mL). VC: viral control. ** *p* < 0.001; **** *p* < 0.00001.

The melting curves were checked and an amplification efficiency of 1.01 was determined. The cDNA concentrations in the viral control and treatments were determined by the formula y = −3.299X + 32.47 (R2 0.998) and are shown in Table 3. The percentage of viral inhibition was calculated by the formula mentioned in the method section. The concentration of 10 µg/mL of TEE inhibited viral replication in 39.83 ± 4.7% and the concentrations of 20 and 30 µg/µL managed to completely inhibit DENV-2 infection (99.9% ± 0.1) (Figure 3).

These results indicate that the action targets of the TEE from *A. hypogaea* in the post-adsorption and penetration stage of DENV-2 are the initial steps of infection, including endosomal pathways or viral stripping, and not the subsequent steps of the multiplication cycle, which include maturation, assembly or viral exocytosis.

The results determined by the molecular technique are consistent with those determined by the lysis plate reduction technique, since 100% viral inhibition was achieved in the treatment with 30 µg/mL of TEE in the same evaluation stage.

In 2018, Makau et al. carried out a study of antiviral activity of the ethanol extract of tegument from *A. hypogaea* [18]. They achieved effective inhibition of influenza virus type A and B, with an IC_50_ corresponding to 1.3 μg/mL in the replication early stages. This shows that the peanut tegument has a great antiviral capacity, since with low concentrations, it achieved the inhibition of DENV-2 and influenza viruses.

On the other hand, the antiviral activity of the TEE from *A. hypogaea* is related to the ethanolic extract’s activity from *Cassia alata* (Fabaceae), since it showed an IC_50_ less than 10 μg/mL and an SI of 32.3 in the post-adsorption stage of the DENV-2 virus [24]

Ramalingam et al. (2018) [13] evaluated the antiviral action mechanism of an ethanolic extract from *Andrographis paniculata* (Burm.f.) Nees (Acanthaceae) against the dengue virus and demonstrated that it acted through the inhibition of viral RNA synthesis. Therefore, this action mode is similar to that shown by the TEE, since both exert antiviral action in the initial steps of infection after adsorption and penetration of the virus. However, the TEE from *A. hypogaea* was able to completely inhibit viral multiplication at 30 μg/mL and the ethanolic extract from *A. paniculata* reduced virus replication by 52% at a 100 μg/mL concentration.

In the section below, we elucidate the probable active compound(s) responsible for the antiviral activity against dengue virus serotype 2. In previous studies on the chemical composition of the TEE of *A. hypogaea*, we identified 74.33 mg GAE/g of total phenols by the Folin–Ciocalteu test (7.43% of its composition) [20]. Makau et al. (2018) [18] also determined the presence of polyphenols in the ethanolic extract of tegument from the peanut plant and they proposed that these components could be responsible for the great inhibitory power that the extract showed against the replication of influenza viruses.

On the other hand, Clain et al. (2019) [25] reported that a polyphenol-rich extract from *Psiloxylon mauritianum* inhibits the early stages of dengue virus and Zika virus infection.

Also, we demonstrated the presence of proanthocyanidins and the absence of phytosterols in the TEE [20]. In the same way, other authors have stated that in peanut skin extracts, the presence of proantocyanidins is due to polymerizations of catechin or epicatechin. The most common proanthocyanidins found in the TEE are A-type procyanidins [26].

Various investigations have indicated that proanthocyanidins possess antiviral capacities. For example, Terlizzi et al. (2016) [27] found that proanthocyanidins are capable of reducing *Herpes simplex* virus. Other studies showed that proanthocyanidins were also active against Rotavirus [28]. In the same way, activity-guided isolation of a methanol leaf extract of Kratom (*Mitragyna speciose*) led to the identification of B-type procyanidin condensed tannins of (-)-epicatechin as virucidal compounds against SARS-CoV-2. The fraction containing condensed tannins exhibited virucidal activity with an EC_50_ value of 8.38 μg/mL and a selectivity index (SI) value > 23.86 [29].

In addition, recent studies of dynamic molecular simulation (in silico, molecular docking) have suggested the proanthocyanidins’ potential to inhibit the coronavirus disease (COVID-19 global pandemic) caused by severe acute respiratory syndrome coronavirus 2 (SARS-CoV-2). The mechanism proposed consists of the ability of the proanthocyanidins/procyanidins to bind with enzymes and proteins involved in the virus replication cycle, the SARSCoV-2 spike protein (S), ACE2 receptor, and the transmembrane serine protein (TMPRSS2), destabilizing the binding between the virus and human cell, and preventing virus replication. In addition, these authors suggest that the activity of proanthocyanidins/procyanidins may alleviate the severity of COVID-19 symptoms and modulate the immune response [30].

Furthermore, we identified the presence of an organic acid, caffeic acid, with a percentage corresponding to 2.46% by HPLC-ESI-MS/MS analysis [20]. Also, other authors have indicated the occurrence of phenolic acids such as vanillic, caffeic, p-coumaric and tartaric acids in peanut skin extracts [26].

In addition, other researchers, such as Alasalvar et al. in 2015, detected caffeic acid in peanuts (2015) [31]. This organic acid is another component that stands out for its great antiviral activity. Flores-Ocelotl et al. (2018) [32] identified caffeic acid in the methanolic extract from *Taraxacum officinale* (Asteraceae), which exerted antiviral activity against the dengue virus. Rodríguez-Ortega et al. (2013) [33] reported the presence of caffeic acid in a chloroform extract of the leaves from *Taraxacum officinale* that was active against the yellow fever virus vaccine strain 17 in the viral adsorption stage.

On the other hand, caffeic acid acts against other important viruses, such as hepatitis B [34], influenza A [35], and *Herpes simplex* viruses [36].

All these studies suggest that the great antiviral activity of the TEE from *A. hypogaea* L. against DENV-2 demonstrated in the present study could be exerted by the compounds found in the extract. This evidence expands on the existing literature that addresses the antiviral activity of plant-derived products, which has enormous potential in different human health scenarios and may share methods of obtaining these products and various mechanisms [37].

## 3. Materials and Methods

### 3.1. Ethanolic Extracts of Peanut

To obtain seed ethanolic extract (SEE) and tegument ethanolic extract (TEE), a simple method of alcoholic extraction reported by García et al. (1990) [38] with modifications was followed. *Arachis hypogaea* L. (peanut) seeds from the cultivar Granoleico were obtained from Criadero El Carmen (General Cabrera, Córdoba, Argentina). The seeds or their tegument were macerated with 80% ethanol for 48 h at 37 °C. Products were consecutively filtered, dried at 37 °C for 3 days, and dissolved in phosphate-buffered saline (PBS). SEE and TEE stocks were filtered first with Whatman Nº 2 filters and then with 0.22 μm pore cellulose acetate filters to be sterilized. A previous phytochemical analysis of the extracts revealed the following compounds in TEE: 74.33 mg/g of phenolic compounds (including 2.46% caffeic acid and 1.39 OD at 550 nm of proanthocyanidins). On the other hand, the SEE mainly included 15.05 mg/g of phenolic compounds, linoleic acid (58.84%), oleic acid (11.31%) and palmitic acid (8.37%), together with other fatty acids at a lower content [20].

### 3.2. Cells and Virus

The Vero cell line derived from African green monkey kidneys (*Cercopithecus aethiops* ATCC CCL-81) was used. Cells were obtained commercially from the Asociación Banco Argentino de Células and were cultured in Eagle-Earle Minimum Essential Medium (MEM of Gibco, Waltham, MA, USA) supplemented with 10% (*v*/*v*) heat-inactivated fetal bovine serum (FBS) (Natocor, Córdoba, Argentina), L-glutamine (30 µg/mL) and gentamicin solution (50 µg/mL) (Sigma-Aldrich, Buenos Aires, Argentina) at 37 °C with CO_2_ (5%).

The dengue virus serotype-2 (DENV-2) New Guinea C strain was used. It was provided by the Universidad de Buenos Aires. The strain was maintained through propagation in Vero cell monolayers and titrated by the lysis plate formation technique [39]. The viral stock was conserved at −80 °C.

### 3.3. Cytotoxicity Assay In Vitro

Vero cells were plated at approximately 3 × 10^4^ per well in the 96-well tissue culture plate and incubated for 24 h. The cell monolayers were treated with increasing concentrations of TEE and SEE (from 5 to 1600 μg/mL). The culture plate was incubated at 37 °C with CO_2_ (5%) for 7 days. The assay was performed in triplicate and cell monolayers with maintenance medium (MM) (containing MEM, 2% FBS) were included as cellular controls.

#### 3.3.1. Neutral Red Uptake (NRU)-Based Assay

After incubation, cells were washed with 200 µL of PBS/well and 200 µL of the NR solution (30 μg/mL in MEM) was added. The plate was incubated at 37 °C for 3 h. Afterward, test solutions were aspirated, the monolayers were washed with PBS and the dye inside the cells was released by extraction with a mixture of acetic acid, ethanol, and water (1:50:49). Finally, the optical density values (O.D.) were measured at 540 nm in a spectrophotometer (Labsystems Multiskan MS, Vantaa, Finland). The relative viability in treatments was expressed as the percentage of captured RN reduction with respect to the control cells. A dose–response curve was constructed to determine the 50% cytotoxic concentration (CC_50_).

#### 3.3.2. 3-(4,5-dimethylthiazol-2-yl)-2,5-diphenyltetrazolium Bromide Metabolism (MTT)-Based Assay

The assay was performed according to the methodology of Mosmann T. et al. reported in 1983 [40]. The Vybrant MTT Cell Proliferation Assay Kit (Molecular Probes Invitrogen Detection Technologies, Eugene, OR, USA) was used. After incubation, solutions were aspirated, cells were washed with 200 µL of PBS and 100 μL of MM without serum and 10 μL of the MTT solution (5 mg/mL of MTT in PBS 0.01 M, pH 7.2) was added to each well. The plate was incubated for 4 h. Then, DMSO was added to solubilize the formazan crystals and the O.D. was measured at 560 nm using a spectrophotometer. The relative viability in treatments was expressed as the percentage of MTT reduction with respect to the cell control. A dose–response curve was constructed to determine the CC_50_.

### 3.4. Activity Antiviral of Ethanolic Extracts

The antiviral activity of the TEE and SEE was evaluated at different stages to elucidate the mechanism of action, including viral adsorption and penetration; viral post-adsorption and penetration; viral pre-treatment; and cell pre-treatment [41,42].

The antiviral action was determined through the reduction in the number of foci viral in the treatment versus the viral control. The percentage of viral inhibition (VI) was calculated as follows: VI (%) = 100 − A/B × 100, where A is the mean of the viral foci number in treated wells with an extract and B is the mean of the viral foci number in control wells (without extract). Then, quantitative RT-PCR (qRT-PCR) was performed to elucidate the target of the most active extract in the dengue virus replication cycle.

#### 3.4.1. Treatment in the Viral Adsorption and Penetration Stage

Vero cells were plated at approximately 2 × 10^5^ cells/well in the 24-well tissue culture plate and incubated for 24 h. When the monolayer was formed, 100 µL of TEE or SEE at a non-cytotoxic concentration in MM and 100 PFU of the virus were added per well and incubated at 37 °C for 1 h. Then, the non-adsorbed virus and extract were discarded, and 1 mL/well of plate medium (MP) containing MEM, FBS (2%), and gentamicin (1%) with ultrapure agarose (0.5%) was added. The system was incubated for 7 days in a humid atmosphere with CO_2_ (5%). Viral (only virus in MM) and cell (MM) controls were included. Treatments were performed in triplicate. After the incubation period, the cells were fixed with formaldehyde (10%) and stained with crystal violet solution (1%) to quantify the viral foci. The calculation of viral inhibition percentages was realized, as above (Section 3.4.).

#### 3.4.2. Treatment in Viral Post-Adsorption and Penetration Stage

Vero cells were plated at approximately 2 × 10^5^ cells/well in the 24-well tissue culture plate and incubated for 24 h. Vero cell monolayers were infected with 100 PFU/well of the virus and were incubated at 37 °C for 1 h. Then, the non-adsorbed virus was eliminated and 1 mL/well of MP with agarose, containing TEE or SEE at a non-cytotoxic concentration, was added. The system was incubated at 37 °C for 7 days in a humid atmosphere with CO_2_ (5%). Viral (only virus in MM) and cell (MM) controls were included. The quantification of viral foci and the calculation of viral inhibition percentages were realized, as detailed above.

#### 3.4.3. Viral Pre-Treatment

To determine the virucidal capacity of the extracts, equal volumes of viral suspension (200 PFU) and the TEE (60 μg/mL) or SEE (600 μg/mL) were mixed in a tube. Cell and viral controls were included (tubes containing virus and MM). All treatments were incubated for 1 h at 37 °C with a humid atmosphere and CO_2_ (5%). Then, the cell monolayers were infected with 200 μL/well of treated and untreated virus in triplicate and incubated for 1 h at 37 °C. Subsequently, the solutions were discarded and 1 mL/well of MP with agarose was added. The system was incubated for 7 days at 37 °C in a humid atmosphere with CO_2_ (5%). Finally, the monolayers were fixed and stained to quantify the number of viral foci and calculate viral inhibition percentages.

#### 3.4.4. Cell Pre-Treatment

Before infection with the virus, cell pre-treatment was performed. It consisted of adding 1 mL/well of MM containing TEE or SEE at a non-cytotoxic concentration to growing cell monolayers, followed by incubation for 1 h at 37 °C. After the extracts were removed, monolayers were washed with PBS and infected with 100 PFU/well. The system was incubated for 1 h at 37 °C. The remaining virus was removed and the cells were incubated with MP and agarose for 7 days at 37 °C in a humid atmosphere with CO_2_ (5%). The viral and cell controls were included. After incubation, all cultures were fixed and stained. From the obtained titles, the percentage of inhibitory action of each extract was calculated.

### 3.5. Quantitative Real-Time PCR Assay (qRT-PCR)

Quantitative RT-PCR was performed to determine the antiviral action mechanism of the most active extract in the viral post-adsorption and penetration stages. First, Vero cells were plated in the 12-well tissue culture plate. Then, cell monolayers were infected with 100 PFU of the virus and were incubated at 37 °C for 1 h. The non-absorbed virus was eliminated and 1 mL/well of MM containing different concentrations (10, 20, and 30 µg/mL) of the extract was added (in triplicate). The system was incubated at 37 °C for 4 days in a humid atmosphere with CO_2_ (5%). After the incubation period, the total intracellular RNA was extracted from viral controls and treatments, using the TRIzol reagent (Invitrogen, Buenos Aires, Argentina). The manufacturer’s instructions were followed with slight modifications. Reverse transcription was carried out with the kit “High-Capacity cDNA Reverse Transcription Kits” from ThermoFisher (RNAase OUT Recombinant Ribonuclease inhibitor, 40 U/μL (Invitrogen, Buenos Aires, Argentina)). The absolute quantification test by real-time PCR was carried out using the “Brilliant III Ultra-Fast SYBR^®^ Green QPCR Master Mix with the Low ROX” kit, (Agilent Technologies, Buenos Aires, Argentina), compatible with Agilent Mx3000P equipment. The reaction mixture contained water, 5 µL (1×) of Brilliant III SYBR Green QPCR Master Mix with Low ROX, 0.35 µL of forward DNF (CT(A,T)TCAATATGCTGAAACGCG) and 0.35 µL of reverse D2R (CGCCACAAGGGCCATGAACAG) primers (10 μM) in a total volume of 9 µL. For each sample, 9 μL of the reaction mixture was taken and 1 μL of cDNA was added. The program consisted of a denaturation stage at 95 °C for 3 min; followed by 40 cycles of amplification (denaturation at 95 °C, 5 s; annealing-extension at 50 °C, 1 min). For absolute quantitation of the viral RNA, a standard curve was established, which was serially diluted to a tenth of cDNA from the DENV-2 viral control (known titer). The percentage of viral inhibition in treatments was determined by the formula 1 − (RNAt/RNAc) × 100, where RNAt is the RNA quantified in treatments and RNAc is the RNA quantified in the viral control [43,44].

### 3.6. Determination of 50% Inhibitory Concentration (IC_50_)

The IC_50_ was determined in the viral post-adsorption and penetration stages. In this way, Vero cells were plated in the 24-well tissue culture plate and after the incubation period, the monolayers were infected with 100 PFU per well of DENV-2 and incubated for 1 h at 37 °C. The residual inoculum was removed; cells were washed with PBS and MEM-0.5% agarose was added together with increasing concentrations of the active extract (1–60 μg/mL). After 7 days at 37 °C, the cultures were fixed, stained, and viral plaques were counted. The IC_50_ was calculated as the extract concentration that reduced the number of PFUs to 50% with respect to the viral control.

### 3.7. Selectivity Index

With this value and the CC_50_ obtained by the cytotoxicity techniques and the IC_50_ obtained in the different treatments, SIs were calculated using the following formula: SI = CC_50_/IC_50_.

### 3.8. Data Analysis

The CC_50_ and IC_50_ were calculated from concentration-effect plots by non-linear regression analysis (Boltzmann sigmoidal) using Graph Pad Prism 6.0. The results account for the mean standard error of the mean values of three different experiments.

## 4. Conclusions

In conclusion, this study demonstrated that the TEE from *A. hypogaea* L. is a potent antiviral that completely inhibits the infection caused by the DENV-2 virus. The extract can be considered a possible antiviral drug of plant origin since it can affect the viral adsorption–penetration stage and the intracellular events of viral replication. In addition, it exerts a virucidal effect because it can act directly on the viral particle, making it defective. On the other hand, the TEE could be used as a prophylactic because it can reduce infection after cell treatment. It would be important, in the future, to investigate the antiviral activity of the pure compounds present in the TEE to understand if the anti-dengue action of the extract is due to any particular component or the synergistic activity of several of these components.

## Figures and Tables

**Table 1:** Viral inhibition percentages for the TEE and SEE from *A. hypogaea* at different stages of the viral cycle in Vero cells.

Extract	Adsorption– Penetration (%)	Post-Adsorption and Penetration (%)	Viral Pre-Treatment (%)	Cell Pre-Treatment (%)
TEE (30 µg/mL)	84.0 ± 1.4	99.9 ± 0.1	92.5 ± 10.6	95 ± 7
SEE (300 µg/mL)	13.7 ± 1.7	51.0 ± 8.6	33.2 ± 5.5	6.0 ± 2.8

**Table 2:** Selectivity indices of TEE from *A. hypogaea* L.

Method	Quotient CC_50_/IC_50_	Selectivity Index
**Neutral Red Uptake**	169/3.47	48.70
**MTT Reduction**	65/3.47	18.73

**Table 3:** Ct values and concentrations of viral cDNA obtained in the viral control and treatments with TEE from *A. hypogaea*.

Sample (µg/mL)	Ct	Viral cDNA Concentration (µg/µL)
**VC**	19.44	2.36
**10**	27.79	1.42
**20**	34.83	0
**30**	35.65	0

## Data Availability

Datasets used can be accessed requesting to the corresponding author.
